# Movable Kidney and Neurasthenia

**Published:** 1905-06-03

**Authors:** 


### MOVABLE KIDNEY AND NEURASTHENIA.

It is natural and correct that whenever in a patient a failure in health is observed to synchronise with some physically recognisable departure from the usual on the part of any organ, an attempt should be made to establish a relationship between the two phenomena. But, unfortunately, in the matter of undue renal mobility, mere induction is far from providing a full conclusion of the matter. In certain dramatic incidents, or rather accidents, of floating kidney, in particular the acute attacks of pain associated with torsion or kinking of the ureter, no doubt remains as to the causal bearing of the renal mobility, but this apart, the complexity of the problem is extreme. Mr. Gilbert Barling[1] has recently published a contribution on the connection between undue renal mobility and certain neurotic phenomena, but before dealing with his views in the matter it will be well to reproduce the reputed symptoms of the condition in the words of Sir Frederick Treves.[2] "Apart from these intense and alarming disturbances (*i.e.* the symptoms of torsion), the symptoms ascribed by various writers to movable kidney may be said to include all those manifold ills which make up the melancholy history of the 'enjoyers of poor health.' There is scarcely an abdominal symptom which has not been placed to the credit of floating kidney. Yet all the classical symptoms of this disorder may be present, and the kidneys be found to be firmly fixed in position." As classical symptoms he suggests "a sense of dragging in the abdomen, a dragging from the loin attended with an undefinable discomfort and feeling of weakness. This discomfort may pass into actual pain. . . Added to this would be certain evidences of abdominal disturbance, such as dyspepsia, flatulence, and constipation. The symptoms are increased by movements, and especially by jolting. . . The relief when in the recumbent position is usually complete. Over and above these come a mysterious exhaustion, a terrorising palpitation, vertigo, irritability of temper, instability of purpose, neuralgias of more or less intense types, insomnia and other symptoms, most of which are described in language of great intensity and vividness by the patient." To this comprehensive list Mr. Barling adds certain cases of migrainous headache, of which he quotes examples. One of these was a lady between 30 and 40 years of age, who was quite incapacitated by migraine. Prolonged rest and recumbency were tried without even temporary benefit, and nephrorrhaphy was practised as a last resource. It was a complete success. Mr. Barling admits that in certain other neurasthenic cases associated with movable kidney (some of them instances of hypochondriasis amounting almost to melancholia), the operation achieved no good result, but he is convinced that the condition is an occasional source of neurasthenia. As an explanation he suggests that the true foundation for the symptoms may be the tension to which the displacement submits the large sympathetic system which lies in close relationship with the kidneys.

In attempting a proper appreciation of this matter the following points present themselves for consideration. In the first place it is insufficient to say that because one person may go through life with a floating kidney which is only revealed by accident, therefore symptoms associated with this condition in another cannot claim the condition as a cause. Idiosyncrasy is an established reality, and the cerebral rendering of painful stimuli undoubtedly varies within wide limits, not only in different people, but in each individual according to varying conditions of general health. In the second place, the exhausting effect of even a slight pain when long continued is familiar to all sufferers from toothache, and cannot be disregarded as a factor in determining the reduction in general well-being so frequently associated with movable kidney; while this reduction, by inclining the patient to magnify her pain, will fully establish a vicious circle. On theoretical grounds, therefore, there is nothing inherently improbable in the causal connection between movable kidney and neurasthenia. On the other hand, it is quite clear that all these so-called classical symptoms may appear without any undue renal mobility, a fact which at once prompts scepticism as to the genuineness of the connection. Further, it may reasonably be argued that a pain which depends upon tension, will disappear when such tension is removed; the pain of uterine prolapse disappears when the strain upon the uterine attachments is removed by recumbency, and it is probably correct to apply the same test to the case of the kidney. Reduced into terms of practice the outcome of this reasoning is as follows. Neurasthenia when associated with a movable kidney does not depend upon the kidney condition unless accompanied by abdominal pain. If associated with abdominal pain neurasthenia may depend upon undue renal mobility, but this is likely to be the case only in the instance of those who obtain relief from the pain by adopting a recumbent position, the kidney, if extremely mobile, being held in position by an appropriate pad.

Such academic argument has obvious limitations, but random surgery in the case of neurasthenics cannot be too highly deprecated: the subjects of the condition are ready to embrace any practical project which promises them relief from a state of very real affliction, a state whose misfortune cannot but be enhanced by the failure of drastic measures. Here is Sir Frederick Treves's opinion. "The treatment that appears to commend itself in the management of a case of movable kidney causing symptoms (short of those of torsion) is the following. Treatment by rest in the recumbent position, with careful feeding, precise attention to the digestive organs, and general massage. The so-called "rest-cure" carried out for a month has not caused the movable kidney to cease to move, but it has rid the patient of her symptoms."

[1] Birmingham Med. Review, March 1905. [2] Pract., Jan. 1905.

